# Protocol for analysis of integrin-mediated cell adhesion of lateral plate mesoderm cells isolated from zebrafish embryos

**DOI:** 10.1016/j.xpro.2021.100428

**Published:** 2021-03-31

**Authors:** Seung-Sik Rho, Eri Oguri-Nakamura, Koji Ando, Kiyotake Yamamoto, Yuki Takagi, Shigetomo Fukuhara

**Affiliations:** 1 Department of Molecular Pathophysiology, Institute of Advanced Medical Sciences, Nippon Medical School, 1-1-5 Sendagi, Bunkyo-ku, Tokyo 113-8602, Japan

**Keywords:** Cell Biology, Cell culture, Cell isolation, Microscopy, Model Organisms

## Abstract

Lateral plate mesoderm (LPM) cells differentiate into various cell types including endothelial and hematopoietic cells. In zebrafish embryos, LPM cells migrate toward the midline along the ventral surfaces of somites during which their cell fate specification depends upon efficient integrin-mediated cell adhesion and migration. Herein, we present a protocol for analysis of integrin-mediated cell adhesion of LPM cells isolated from zebrafish embryos. This allows the study of the molecular mechanisms underlying integrin activation required for LPM cell fate specification.

For complete details on the use and execution of this protocol, please refer to Rho et al. (2019).

## Highlights

• Zebrafish breeding and preparation of the LPM cells dissociated from the embryos
• Protocol for LPM cell adhesion to the fibronectin-coated dish
• Imaging the LPM cells adhering to the fibronectin-coated dish
• Analysis of cell spreading and focal adhesion formations in the LPM cells

## Graphical abstract

**Figure:**
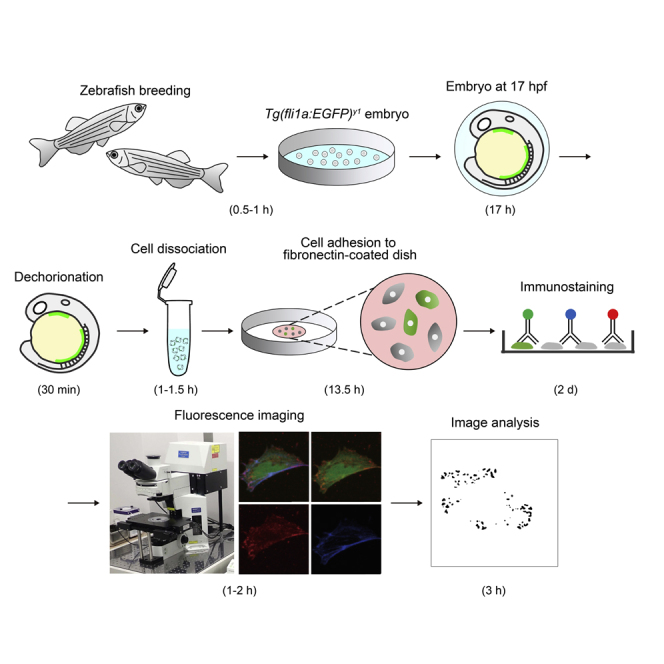

## Before you begin

### Zebrafish

**Figure 1:**
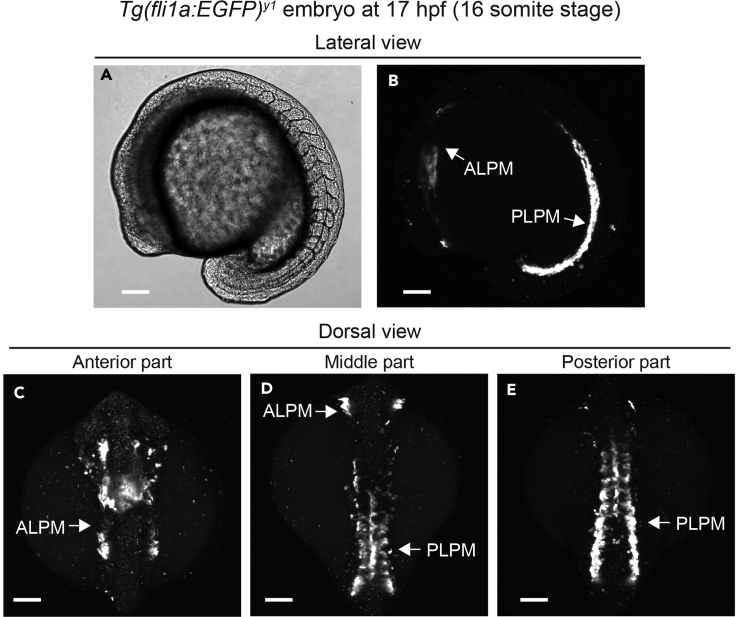
Visualization of LPM cells in the *Tg(fli1a:EGFP)*^*y1*^ zebrafish embryos at 17 hpf (A and B) DIC (A) and confocal stack fluorescence (B) images showing lateral view of the *Tg(fli1a:EGFP)*^*y1*^ embryo at 17 hpf (16 somite stage). Note that EGFP fluorescence labels the anterior LPM and posterior LPM (B). (C–E) Confocal stack fluorescence images showing dorsal view of the *Tg(fli1a:EGFP)*^*y1*^ embryo. Anterior (C), middle (D), and posterior (E) parts of the embryos are shown. ALPM, anterior lateral plate mesoderm; PLPM, posterior lateral plate mesoderm. Scale bars, 100 μm.

To visualize the LPM cells, we have used the *Tg(fli1a:EGFP)*^*y1*^ zebrafish line in which EGFP is expressed in LPM cells during somitogenesis and in endothelial cells at a later stage (Lawson and Weinstein, 2002) (Figure 1). The *Tg(fli1a:EGFP)*^*y1*^ zebrafish are intercrossed to obtain embryos exhibiting strong EGFP fluorescence in the LPM cells. In our study, we also used *rap1b*^*ncv124*^ mutant fish with the background of the *Tg(fli1a:EGFP)*^*y1*^ zebrafish line to investigate the role of Rap1b in integrin-mediated cell adhesion of LPM cells (Rho et al., 2019).

**CRITICAL:** Using embryos exhibiting strong EGFP fluorescence in LPM cells is important for obtaining clear images of the LPM cells adherent to the fibronectin-coated culture dish. The paper describing the generation of *Tg(fli1a:EGFP)* zebrafish line indicated that the level of EGFP expression correlates with transgene copy numbers and that the *Tg(fli1a:EGFP)*^*y1*^ line used in our study carries more than 25 copies of transgene (Lawson and Weinstein, 2002). Thus, using the zebrafish lines carrying high copy numbers of the transgene provides clear images.

***Note:*** In the *Tg(fli1a:EGFP)*^*y1*^ line, expression of EGFP in LPM cells and endothelial cells does not alter cellular physiology and behavior, because hematopoiesis and vascular development normally occur in this transgenic line (Rho et al., 2019; Lawson and Weinstein, 2002).

***Alternatives:*** Instead of the *Tg(fli1a:EGFP)*^*y1*^ zebrafish line, you may be able to use transgenic zebrafish lines expressing various types of fluorescence proteins under control of the *fli1a* promoter to visualize cell morphology and cellular structures such as the actin cytoskeleton. For instance, using *Tg(fli1a:Myr-EGFP)* and *Tg(fli1a:Lifeact-mCherry)* lines allows us to image LPM cell morphology and the actin cytoskeleton, respectively (Wakayama et al., 2015). However, embryos exhibiting strong fluorescence must be used to obtain clear images. It may also be possible to use zebrafish mutants and morphants to investigate the mechanisms of integrin-mediated cell adhesion and migration of LPM cells.

### Fibronectin-coated glass base dish

**Timing: 1 day**

Coat the 35-mm-diameter glass-base dishes with 200 μL of 10 μg/mL fibronectin in PBS at 4°C and leave them for 12–16 h. Wash the dishes with PBS or sterile water before use.

***Note:*** Fibronectin-coated dishes washed with sterile water can be stored at 2–8°C under sterile conditions for several days.

## Key resources table

**Table:** 

REAGENT or RESOURCE	SOURCE	IDENTIFIER
**Antibodies**		

Rabbit anti-GFP	Thermo Fisher Scientific	Cat#A11122; RRID: AB_221569
Mouse anti-vinculin	Sigma-Aldrich	Cat#V9131; RRID: AB_477629
Alexa Fluor 488-conjugated goat anti-rabbit IgG	Thermo Fisher Scientific	Cat#A-11034; RRID: AB_2576217
Alexa Fluor 546-conjugated goat anti-mouse IgG	Thermo Fisher Scientific	Cat#A-11030; RRID: AB_144695

**Chemicals, peptides, and recombinant proteins**		

Pronase	Roche	Cat#10165921001
Liberase	Sigma-Aldrich	Cat#05401119001
Leibovitz’s L-15 medium	Gibco	Cat#11415-064
Fibronectin	Wako Pure Chemical	Cat#063-05591
10% Formaldehyde	Sigma-Aldrich	Cat#11-0735
Bovine serum albumin (BSA)	Sigma-Aldrich	Cat# A2153
Alexa Fluor 633-conjugated phalloidin	Invitrogen	Cat#A22284

**Experimental models: organisms/strains**		

Zebrafish: *rap1b*^*ncv124*^	(Rho et al., 2019)	N/A
Zebrafish: *Tg(fli1a:GFP)*^*y1*^	(Lawson and Weinstein, 2002)	N/A

**Software and algorithms**		

ImageJ software	National Institutes of Health	http://imagej.nih.gov/ij/download.html
FluoView ASW4.2 viewer	Olympus	http://www.olympus-lifescience.com/en/downloads/
GraphPad Prism 5 software	GraphPad Software Inc	http://www.graphpad.com/scientific-software/prism/E

**Other**		

35-mm-diameter glass-base dish	Asahi Techno Glass	Cat#3961-035
Sea salt	REI-SEA; IWAKI	Cat#Rei-Sea marine II
Dulbecco's phosphate-buffered saline (DPBS)	Thermo Fisher Scientific	Cat#14040133
Fetal bovine serum (FBS)	Biowest	Cat#S1810-500
Penicillin-streptomycin-amphotericin B Suspension (×100) (antibiotic-antimycotic solution)	FUJIFILM Wako	Cat#161-23181
Cell strainer, size 100-μm	Corning	Cat# CLS431752
Fluorescence stereomicroscope	Olympus	Model: SZX16
FluoView FV1000 confocal upright microscope	Olympus	Model: FV1000
Water-immersion 60× lens	Olympus	Model: LUMFL N 60×/1.10 W

***Alternatives:*** Cell culture grade fibronectin can be obtained from the alternative companies such as Sigma-Aldrich. The 35-mm-diameter dishes with 12-mm diameter glass bottom can also be purchased from the alternative companies such as Thermo Scientific Nunc.

***Alternatives:*** Although we used FluoView FV1000 confocal upright microscope to image the LPM cells, equivalent confocal microscope can be used.

## Materials and equipment

**Table:** E3 medium: 50× stock solution

Reagent	Amount	Final concentration
NaCl	14.6 g	250 mM
KCl	0.63 g	8.5 mM
CaCl_2_∙2H_2_O	2.43 g	16.5 mM
MgSO_4_∙7H_2_O	4.07 g	16.5 mM
RO water	Up to 1 L	
Total	n/a	1 L

Adjust pH to 7.2 with 0.1 M NaOH solution.

To prepare 1× E3 medium, add 20 mL of the 50× stock solution to 980 mL of RO water. Final working solution: 5 mM NaCl, 0.17 mM KCl, 0.33 mM CaCl_2_, 0.33 mM MgSO_4_.

**Table:** 0.03% sea salt

Reagent	Amount	Final concentration
Sea salt	0.3 g	
RO water	Up to 1 L	
Total	n/a	1 L

**Table:** Pronase stock solution

Reagent	Amount	Final concentration
Pronase	0.5 g	10 mg/mL
E3 medium	Up to 50 mL	
Total	n/a	1 L

Make 1 mL/tube aliquots in 1.5 mL-Eppendorf tubes and store at −20°C

To prepare working solution, add 1 mL of 10 mg/mL Pronase to 9 mL of 0.03% sea salt solution.

**Table:** Liberase: 100× stock solution

Reagent	Amount	Final concentration
Liberase	5 mg	5 mg/mL
Sterile water	1 mL	
Total	n/a	5 mL

**CRITICAL:** Directly add 1 mL of sterile water into the vial containing 5 mg Liberase and gently agitate the vial at 4°C until 30 min. Make the aliquots and store at −20°C. Do not repeat freeze/thaw cycles.

To prepare working solution of 50 μg/mL Liberase in DPBS, add 50 μL of 100× stock solution to 450 μL DPBS.

**Table:** Liberase termination solution

Reagent	Amount	Final concentration
FBS	0.2 mL	2%
PBS	9.8 mL

**Table:** Culture media

Reagent	Amount	Final concentration
Leibovitz’s L-15 medium	42.0 mL	
FBS	7.5 mL	15%
1M CaCl_2_ solution	40 μL	0.8 mM
Antibiotic-Antimycotic Solution (×100)	0.5 mL	100 U/mL Penicillin G, 100 μg/mL Streptomycin, 0.25 μg/mL Amphotericin B
Total	n/a	50 mL

**Table:** Fixative solution

Reagent	Amount	Final concentration
10% Formaldehyde	2 mL	2%
PBS	8 mL	
Total	n/a	10 mL

**Table:** Permeabilization solution

Reagent	Amount	Final concentration
20% Triton X-100	0.025 mL	0.05%
PBS	9.975 mL	
Total	n/a	10 mL

**Table:** Blocking solution

Reagent	Amount	Final concentration
BSA	2 g	4%
PBS	50 mL	
Total	n/a	50 mL

## Step-by-step method details

### Embryo preparation

**Timing: 19 h**

Embryos obtained by intercrossing the *Tg(fli1a:EGFP)*^*y1*^ zebrafish line are collected and grown until 17 h post-fertilization (hpf) (about the 16 somite stage).

**Figure 2:**
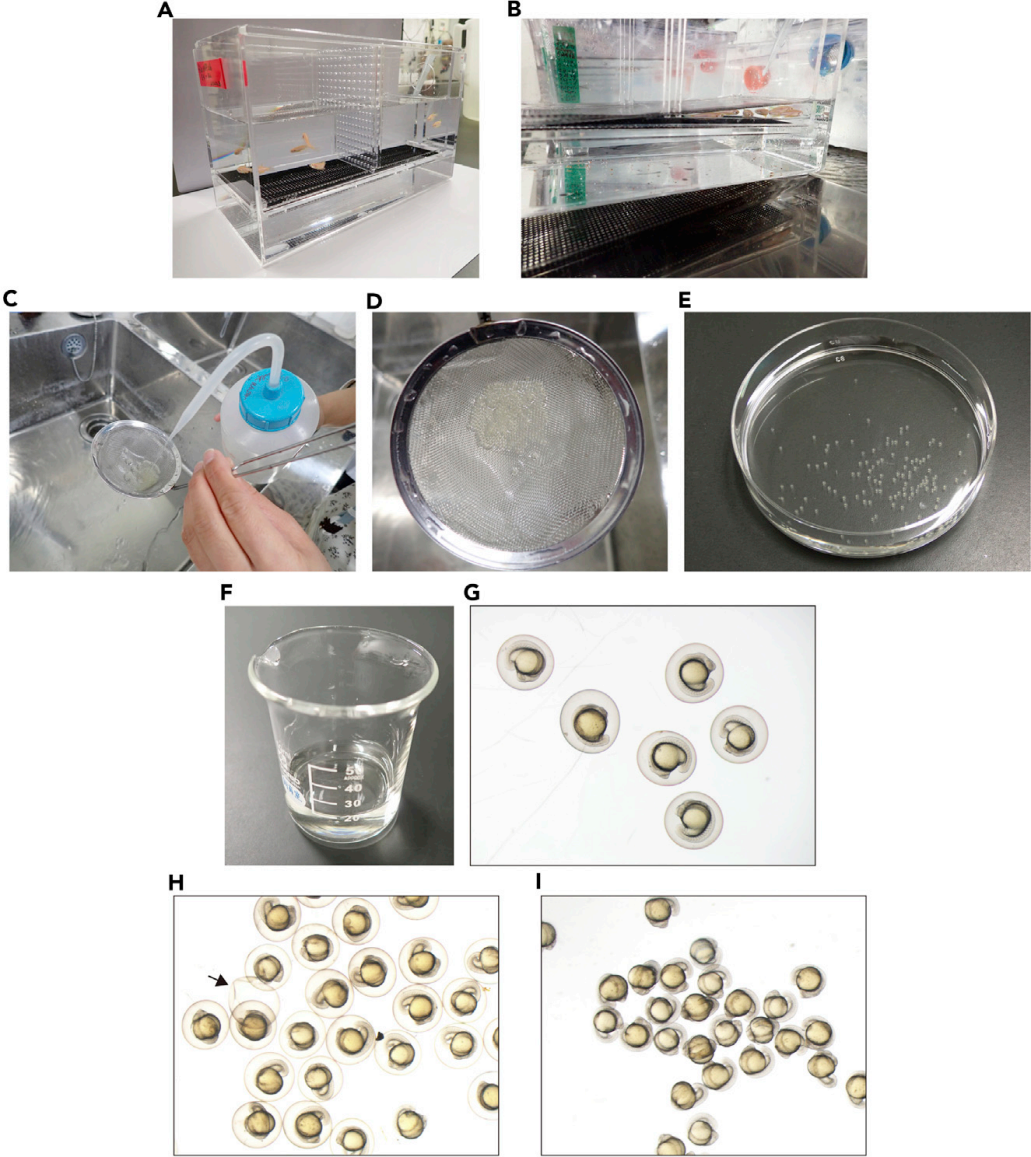
Preparation of the dechorionated zebrafish embryos at 17 hpf (A) Set up zebrafish to breed in the separate breeding tank (step 1). (B) Initiate breeding by removing the divider separating the male and female fish (step 2). (C) Rinse the embryos on the strainer with 0.03% sea salt solution (step 5). (D) Cleaned embryos on the strainer. (E) The embryos transferred into a 10-cm petri dish containing E3 medium (step 6). (F and G) The 17 hpf embryos transferred into a beaker containing E3 medium (step 8b). (H) The embryos treated with the Pronase solution (step 8d). Note that the chorions start to break open (arrow). (I) Dechorionated embryos in a 10-cm petri dish containing E3 medium (step 8g).

***Note:*** To stimulate good quality egg production, the day-night light cycle (14 h light/10 h dark cycle) is controlled with an automatic timer, because zebrafish reproduction depends on the light cycle.

1. Transfer the *Tg(fli1a:EGFP)*^*y1*^ zebrafish into the breeding tank in which the male and female fish are separated by a divider during the afternoon or evening (Figure 2A).

2. When the light comes on the following morning, remove the divider separating the male and female fish to initiate breeding (Figure 2B).

***Note:*** Breeding tanks have a removable insert with holes to prevent adult fish from eating their eggs.

***Note:*** Reducing the amount of water inside the tank facilitates zebrafish breeding.

3. Remove the adult fish 15–30 min after the beginning of spawning.
4. Collect the embryos by pouring the water and the embryos through a mesh tea strainer.
5. Rinse the embryos on the strainer well with 0.03% sea salt solution (Figures 2C and 2D).
6. Wash the embryos off into a 10-cm petri dish using E3 medium (Figure 2E).
7. Keep the embryos in petri dishes containing E3 medium at a density of less than 30 embryos per dish until 17 hpf at 28°C.

***Note:*** Do not incubate the embryos at a density of more than 30 embryos per dish to prevent developmental delay.

### Preparation of the cells dissociated from the embryos

**Timing: 1.5–2 h**

The *Tg(fli1a:EGFP)*^*y1*^ embryos are dissociated into a single cell suspension. Screen the embryos with a fluorescence stereo microscope in advance, selecting the brightest embryos.

8. Dechorionation of zebrafish embryos
  a. At 17 hpf, transfer the *Tg(fli1a:EGFP)*^*y1*^ zebrafish embryos into glass beakers (Figures 2F and 2G).
  b. Incubate the embryos in the Pronase solution at 20–25°C for 5–10 min.
  c. During the incubation, gently mix the embryos once per minute and check the condition of the chorions under a microscope.
  d. Wash the embryos thoroughly with E3 medium several times when the chorions start to break open (Figure 2H).
  e. Pipet the embryos back and forth in E3 medium using plastic Pasteur pipette to remove the chorions.
  f. Collect the dechorionated embryos in the 10-cm petri dish containing E3 medium (Figure 2I).

9. Enzymatic dissociation of the embryos
  a. Transfer fifteen embryos into a 2 mL-Eppendorf tube.
    ***Note:*** If possible, select embryos at the same stage by counting the number of somites.
  b. Wash the embryos with 1 mL of PBS once.
  c. Incubate the embryos with 500 μL of 1× Liberase solution at 37°C for 1 h.
    ***Note:*** During the incubation, gently mix the embryos every 20 min using a P1000 single channel Pipette.
  d. After the incubation, add 1 mL of Liberase termination solution to the tube and mix to terminate the enzymatic reaction.
  e. Dissociate the embryonic cells by gentle pipetting using a P1000 Pipette.
  f. Filter the dissociated cells through a 100-μm cell strainer.
  g. Collect the cells in new 2 mL-Eppendorf tubes.
  h. Pellet the cells by centrifugation at 1,600 rpm (approximately 250 × *g*) for 3 min at 15–25°C.
  i. Remove the supernatant.
  j. Resuspend the cells in 1 mL of DPBS.

### Adhesion of LPM cells dissociated from the embryos to the fibronectin-coated dish

**Timing: 13.5 h**

The LPM cells dissociated from the *Tg(fli1a:EGFP)*^*y1*^ zebrafish embryos are allowed to adhere to the fibronectin-coated dish.

10. Pellet the cells suspended in DPBS (from step 9-j) by centrifugation at 1,600 rpm (approximately 250 × *g*) for 3 min at 15–25°C.
11. Remove the supernatant.
12. Resuspend the cells in 1 mL of Leibovitz’s L15 culture medium.
13. Plate 200 μL of the resuspended cells onto the fibronectin-coated glass bottom dish (12-mm diameter glass bottom).

***Note:*** The cells derived from the embryos contain not only GFP-positive LPM cells but also GFP-negative cells.

***Optional:*** While the GFP-positive LPM cells can be isolated using a fluorescence-activated cell sorter, this step might result in cell damage.

14. Incubate the cells, thereby allowing them to adhere to the fibronectin-coated dish, at 28°C for 13 h.

### Staining of the LPM cells adhering to the fibronectin-coated dish

**Timing: 2 days**

The cells adhering to the fibronectin-coated dish are stained with rabbit anti-GFP and mouse anti-vinculin antibodies followed by Alexa Fluor 488-conjugated anti-rabbit and Alexa Fluor 564-conjugated anti-mouse secondary antibodies and with Alexa Fluor 633-conjugated phalloidin to analyze cell spreading and the formation of focal adhesions in the LPM cells.

15. After the incubation, gently wash the adherent cells with PBS twice.
16. Fix the cells with fixative solution for 20 min at 15–25°C.
17. Permeabilize the cells with permeabilization solution for 30 min at 4°C.
18. Wash the cells with PBS three times, briefly.
19. Incubate the cells with blocking solution for 90 min at 15–25°C.
20. Incubate the cells with rabbit anti-GFP antibody (1:300 dilution) and mouse anti-vinculin antibody (1:300 dilution) dissolved in PBS at 4°C for 12–16 h.
21. Wash the cells with PBS three times for 10 min each.
22. Label the proteins reacting with primary antibodies by incubating the cells with PBS containing Alexa Fluor 488-conjugated goat anti-rabbit IgG antibody (1:1000 dilution) and Alexa Fluor 564-conjugated goat anti-mouse IgG antibody (1:1000 dilution) for 2 h at 15–25°C.

**CRITICAL:** Execute steps 22–26 in the dark.

23. Wash the cells with PBS six times for 10 min each.
24. Incubate the cells with PBS containing Alexa Fluor 633-conjugated phalloidin (1:1000 dilution) for 2 h at 15–25°C to visualize filamentous actin.
25. Wash the cells with PBS three times for 10 min each.
26. Submerge the cells with PBS.

**Pause point:** The stained cells can be stored in the dark at 4°C for several days.

### Fluorescence imaging of the LPM cells adhering to the fibronectin-coated dish

**Timing: 1–2 h**

**Figure 3:**
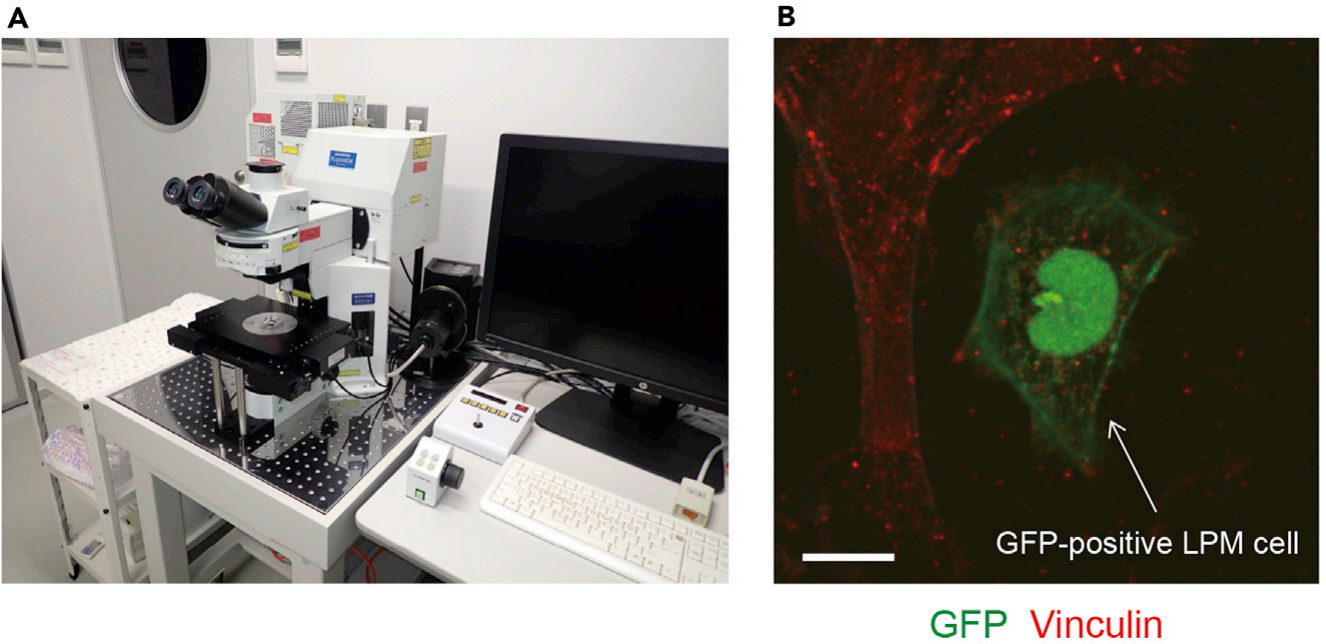
LPM cells adhering to the fibronectin-coated dish (A) FluoView FV1000 confocal upright microscope system. (B) The cells derived from 17 hpf embryos were left to adhere to the fibronectin-coated dish for 13 h and then stained with anti-GFP (green) and anti-vinculin (red) antibodies. Arrow indicates GFP-positive LPM cell. Scale bar, 10 μm.

Fluorescence images of the LPM cells adhering to the fibronectin-coated dish are obtained with a FluoView FV1000 confocal upright microscope system equipped with a water-immersion 60× lens (LUMFL N 60×/1.10 W), 473-, 559-, and 635-nm laser lines, and a GaAsP photomultiplier tube controlled with FluoView ASW software (Figure 3A).

27. We set the confocal imaging parameters as follows, but you will need to optimize these to achieve reasonable signal-to-noise ratio in your image without saturating the detector, as well as taking care to use Nyquist sampling:
  a. Scanning speed; 10 μs/pixel
  b. Sampling; 640 × 640 pixels, 0.094 μm/pixel
  c. Zoom; 3.5× for a FOV of 60 × 60 μm
  d. Laser power; 473 nm 1.0%–1.5%, 559 nm 4.0%–5.0%, 635 nm 1.0%–2.5%
    ***Note:*** 100% laser power of 473 nm, 559 nm, and 635 nm lasers are equivalent to 15 mW, 15 mW, and 20 mW, respectively.
  e. Pinhole diameter; 150–250 μm
  f. Step size; 0.47 μm
  g. Filter mode; Line Kalman 2
  h. Scanning mode; Sequential line scanning
  i. PMT channel parameter settings; HV 450–650, Gain 1×, Offset -5–5

28. Define a region where GFP-positive LPM cells are present.

***Note:*** Select the GFP-positive LPM cells that are not in contact with the surrounding GFP-negative cells.

29. Acquire the fluorescence image volumes of Alexa Fluor 488 (GFP, wavelength range: 485–545 nm), Alexa Fluor 546 (vinculin, wavelength range: 570–625 nm), and Alexa Fluor 633 (F-actin, wavelength range: 655–755 nm) (Figure 3B).

### Quantitative analysis of cell spreading and focal adhesion formations in the LPM cells adhering to the fibronectin-coated dish

**Timing: 3 h**

**Figure 4:**
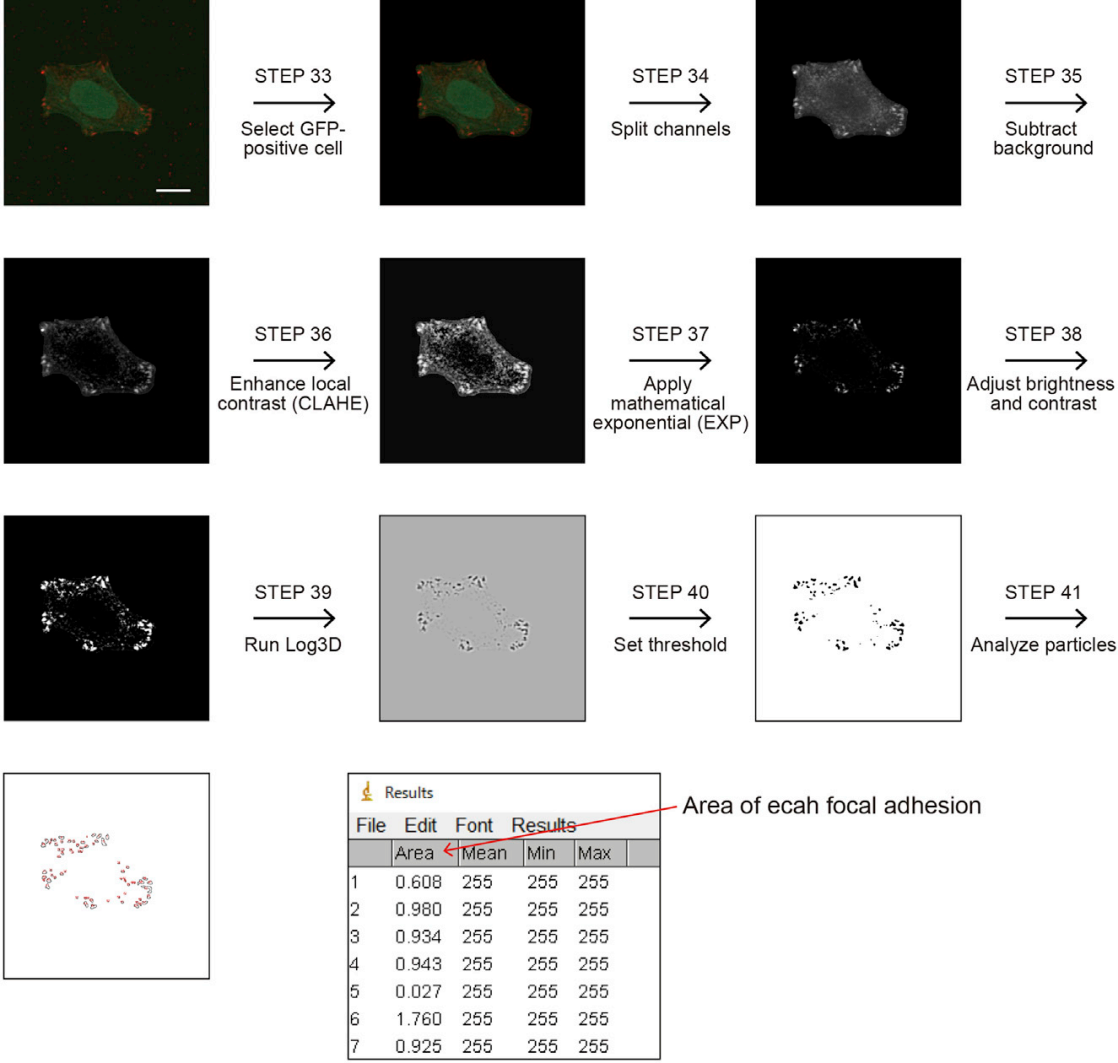
Sequence of image processing by ImageJ for analyzing the number and size of focal adhesions Scale bar, 10 μm.

Cell spreading area and the number and size of focal adhesions were analyzed using ImageJ software according, basically, to the protocol described previously (Horzum et al., 2014) (Figure 4).

30. Open the images using ImageJ software (Schneider et al., 2012).

***Note:*** Install the Bio-Formats in ImageJ to open Olympus OIF files from the link https://www.openmicroscopy.org/bio-formats/downloads/ (Linkert et al., 2010).

31. Create the Z-projections.
  a. [Image]→[Stacks]→[Z Project]

32. (Optional) If size information is lost when opening the stacked Images, set the scale using the image with a calibration scale.
  ***Note:*** Checking the [Show Info] ([Image]→[Show Info]) let you know whether the size information is lost.
  a. Draw a straight line that defines a known distance on your calibration image using the “straight line selection” tool.
  b. [Analyze]→[Set Scale]
  c. Fill in the “Known distance” without units.
  d. Define the units of length in the “Unit of Length” field.
  e. Click on “Global” so that this calibration applies to all images that you open in this ImageJ session.
  f. Click “OK”.

33. Select the GFP-positive cell for analysis.
  a. Draw the outline of the GFP-positive cell by using Freehand selections.
  b. [Edit]→[Clear Outside]
34. Separate images for each channel.
  a. [Image]→[Color]→[Split Channels]
  b. Select the Alexa Fluor 546 image (vinculin).
35. Subtract background.
  a. [Process]→[Subtract Background]
  b. Chose the “Sliding Paraboloid” option with the “Rolling ball radius” set to 50 pixels.
36. Enhance the local contrast of the image.
  a. [Plugin]→[CLAHE]
  b. Set the values as follows; block size = 19, histogram bin = 256, maximum slope = 6.

***Note:*** To execute this step, “CLAHE plugin” needs to be installed in ImageJ from the link https://imagej.nih.gov/ij/plugins/clahe/index.html.

37. Minimize the background.
  a. [Process]→[Math]→[Exp]
38. Adjust brightness and contrast.
  a. [Image]→[Adjust]→[Brightness/Contrast]
  b. Click “Auto” and “Apply”.
39. Run Log3D (Laplacian of Gaussian) filter.
  a. [Plugin]→[Log3D]
  b. Define the size of the Log3D filter as sigma *X* = 3 and sigma *Y* = 3.

***Note:*** To execute this step, “Log3D plugin” needs to be installed in ImageJ from the link http://bigwww.epfl.ch/sage/soft/LoG3D/ (Sage et al., 2005).

40. Set the threshold.
  a. [Image]→[Adjust]→[Threshold]
  b. Click “Apply”.
41. Run analyze particles.
  a. [Analyze]→[Analyze Particles]→[Threshold]
  b. Set the parameters as follows; Size = 0–infinity, Circularity = 0.00–0.99.
  c. Select the “Show: Outlines” option.
  d. Check “Display results” and “Summarize”.

## Expected outcomes

**Figure 5:**
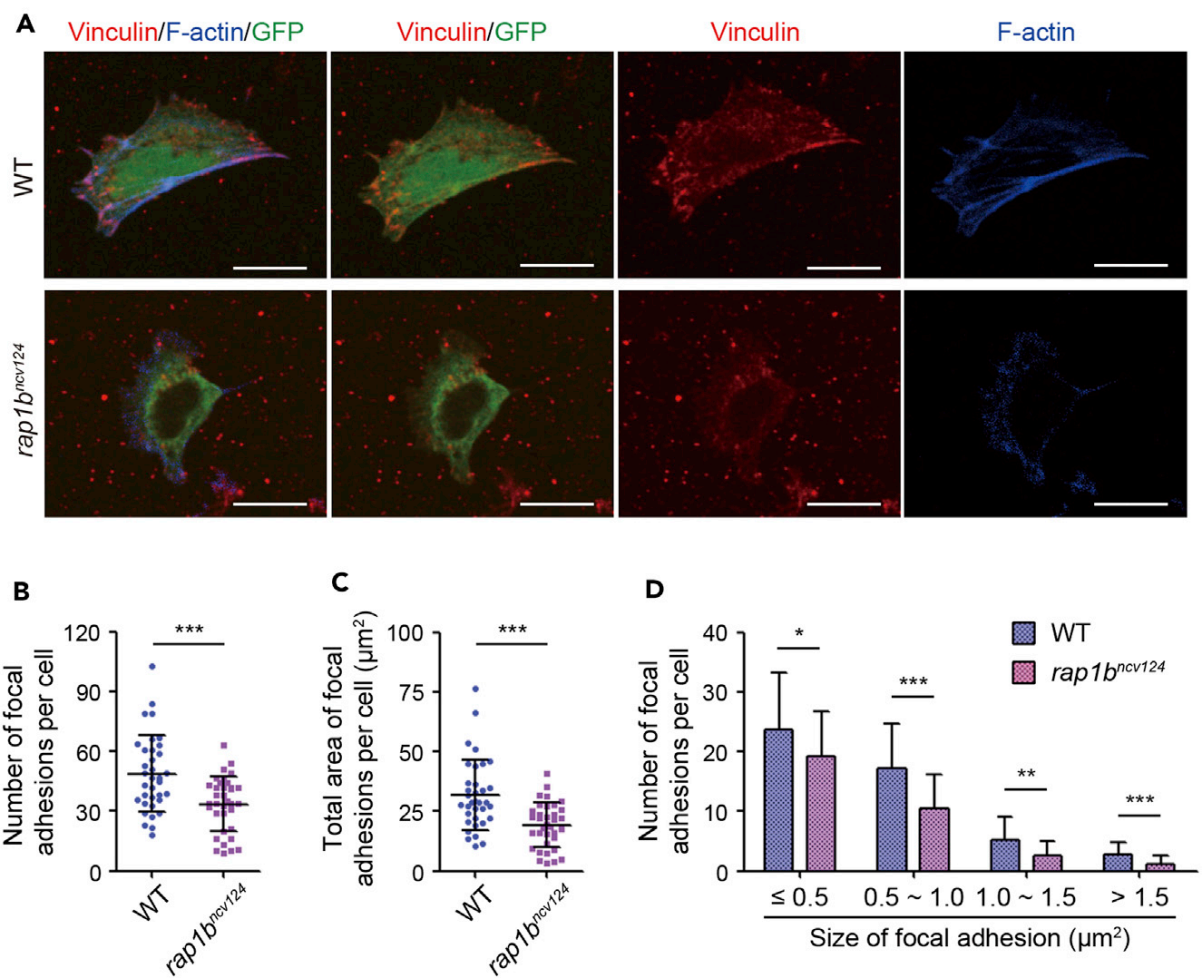
Rap1b regulates integrin-mediated adhesion of LPM cells to fibronectin-coated dishes (A) Cells dissociated from 17 hpf wild type (upper) and *rap1b*^*ncv124*^ (lower) *Tg(fli1a:GFP)*^*y1*^ embryos. Merged images of GFP (green)/vinculin (red)/F-actin (blue), those of GFP (green)/vinculin (red), and vinculin (red) and F-actin (blue) images are shown, as indicated. Scale bars, 10 μm. (B) Number of vinculin-marked focal adhesions per cell, as observed in (A). (C) Total area of vinculin-marked focal adhesions per cell, as observed in (A). (D) Number of vinculin-marked focal adhesions, as observed in (A). Data are shown as means ± s.d. ∗p < 0.05; ∗∗p < 0.01; ∗∗∗p < 0.001. (B)-(D) reuse parts of a figure from Rho et al. (Rho et al., 2019).

If this protocol is followed, most of the GFP-positive LPM cells derived from the *Tg(fli1a:EGFP)*^*y1*^ zebrafish embryos form integrin-mediated focal adhesions and efficiently adhere to the fibronectin-coated dish after 13 h of culture, as shown in Figure 5A (Rho et al., 2019). The cell spreading area of the GFP-positive LPM cells on the fibronectin-coated dish is 327.4 ± 17.09 μm^2^ (n=35). The number of focal adhesions per cell and total area of focal adhesions per cell are 48.77 ± 3.24 and 31.84 ± 2.489 μm^2^, respectively (n=35) (Figures 5B and 5C). Thus, this protocol enables us to quantitatively analyze formation of integrin-mediated cell adhesion of LPM cells derived from zebrafish embryos.

By utilizing this protocol, we previously investigated the role of Rap1b, a small GTPase that belongs to the Ras superfamily, in integrin-mediated cell adhesion of LPM cells involved in hematopoietic stem cell development (Figure 5) (Rho et al., 2019). In this study, we found that LPM cells derived from *rap1b*^*ncv124*^ mutant embryos exhibited less spreading on the fibronectin-coated dish than the wild type cells. We also showed the number and size of focal adhesions to be significantly smaller in *rap1b*^*ncv124*^ mutant-derived LPM cells than in wild type cells (Figures 5B and 5C). Notably, *rap1b*^*ncv124*^ mutant-derived LPM cells exhibited a significant decrease in the number of mature focal adhesions (Figure 5D). By performing the experiments using this protocol, we have succeeded in demonstrating that Rap1b induces formation and maturation of integrin-based focal adhesions to promote LPM cell adhesion to fibronectin.

## Limitations

This protocol uses the *Tg(fli1a:EGFP)*^*y1*^ zebrafish line to identify LPM cells plated onto fibronectin-coated dishes. However, the *fli1a* promoter drives gene expression in both anterior and posterior LPM cells. Therefore, this protocol does not allow us to distinguish whether GFP-expressing cells are anterior or posterior LPM cells. This limitation might be overcome by cutting the embryos into the anterior and posterior parts, although it takes time because 17 hpf embryos are very small.

The mutant zebrafish can be used to investigate the role of a gene of interest in integrin-mediated cell adhesion of LPM cells. However, wild type and mutant fish need to be intercrossed to obtain wild type LPM cells and those derived from the mutants, respectively, because we are unable to perform genotyping of each embryo at 17 hpf before cell dissociation. Alternatively, the embryos injected with control morpholino oligonucleotide and with that targeting a gene of interest can be used, although it would be necessary to confirm that observed effects are specific to the mutants used.

## Troubleshooting

### Problem 1

Insufficient number of embryos collected (step 2).

### Potential solution

Maintaining adult zebrafish in a healthy condition is important to get good quality and large number of embryos. Therefore, it is advised to feed the fish more food than usual for several days before breeding. In addition, the breeding fish should be between 3 and 12 months of age for production of large number of embryos, although zebrafish reach sexual maturity in 2 to 3 months.

### Problem 2

Abnormal development and low fertilization rates (step 7).

### Potential solution

As mentioned above, use of adult zebrafish in a healthy condition is important to get good quality embryos. Thus, refer to the potential solution for “problem 1”. In addition, keeping zebrafish embryos in a clean environment is necessary for the normal development. Therefore, when collecting the embryos, rinse them on the strainer thoroughly to remove feces and residual food (step 5) (Figure 2C). In addition, remove the abnormal embryos from the petri dish during the incubation.

### Problem 3

Insufficient dechorionation of zebrafish embryos (step 8).

### Potential solution

Incubating the embryos with the Pronase solution at 28°C instead of 20–25°C facilitates the dechorionation. But, be careful to prevent the embryos to be damaged. For that, carefully check the condition of the chorions during the incubation and control the incubation time. Alternatively, the chorions can be manually removed with fine forceps. But, take great care not to damage the embryos, because they are fragile.

### Problem 4

Drying of cells on the glass-base dish by evaporation of Leibovitz’s L-15 medium (step 13).

### Potential solution

Incubation of the cells plated onto a glass-base dish in a fish incubator at 28°C may result in evaporation of the medium. To prevent medium evaporation and drying of the cells, place the dishes inside a 150 mm petri dish in which papers soaked with distilled water have been placed. Careful addition of the medium to the dish 9 h after the plating of cells is also feasible.

### Problem 5

Too few GFP-positive cells in field of view (step 28).

### Potential solution

The cells isolated from the embryos showing weak GFP fluorescence may be difficult to detect. Therefore, when selecting the *Tg(fli1a:EGFP)*^*y1*^ zebrafish embryos at 17 hpf under the fluorescence stereomicroscope in step 8, it is advised that the embryos exhibiting strong GFP fluorescence be collected. This allows LPM cells expressing a high level of GFP to be obtained.

### Problem 6

Weak fluorescence signal obtained from the stained cells (step 29).

### Potential solution

Incubation with Alexa Fluor-labeled secondary antibodies at 4°C for 12–16 h instead of that for 2 h at 15–25°C may increase fluorescence signal (step 22). In addition, staining of the cells with higher concentration of primary antibodies may also improve the signal (step 20).

## Resource availability

### Lead contact

Further information and requests for resources and reagents should be directed to and will be fulfilled by the lead contact, Shigetomo Fukuhara (s-fukuhara@nms.ac.jp).

### Materials availability

The *Tg(fli1a:EGFP)*^*y1*^ zebrafish line, which was originally developed by Nathan Lawson and Brant Weinstein (Lawson and Weinstein, 2002), used in this study can be obtained from the Zebrafish International Resource Center (https://zebrafish.org/fish/lineAll.php). The *rap1b*^*ncv124*^ mutant zebrafish will be deposited at the National BioResource Project Zebrafish in Japan (https://shigen.nig.ac.jp/zebra/index_en.html).

### Data and code availability

This study generated neither any unique datasets nor code.
